# FLCN Regulates HIF2α Nuclear Import and Proliferation of Clear Cell Renal Cell Carcinoma

**DOI:** 10.3389/fmolb.2020.00121

**Published:** 2020-07-28

**Authors:** Xuyang Zhao, Yadong Ma, Jie Cui, Haiyang Zhao, Lei Liu, Yueyuan Wang, Pengxiang Min, Lin Zhang, Yongchang Chen, Jun Du, Yujie Zhang, Luo Gu

**Affiliations:** ^1^Department of Biochemistry and Molecular Biology, Nanjing Medical University, Nanjing, China; ^2^Jiangsu Key Laboratory of Cancer Biomarkers, Prevention and Treatment, Collaborative Innovation Center for Cancer Personalized Medicine, Nanjing Medical University, Nanjing, China; ^3^Department of Physiology, Nanjing Medical University, Nanjing, China; ^4^Department of Physiology, Xuzhou Medical University, Xuzhou, China; ^5^State Key Laboratory of Reproductive Medicine, Department of Anatomy, Histology and Embryology, Nanjing Medical University, Nanjing, China; ^6^Department of Physiology, Jiangsu University, Zhenjiang, China

**Keywords:** FLCN, mTORC2, HIF2α, nuclear import, cell proliferation

## Abstract

**Aims and Hypothesis:** This study aims to explore the specific molecular mechanism of folliculin (FLCN)-induced proliferation, migration, and invasion in clear cell renal cell carcinoma (ccRCC) and to investigate the relationship of FLCN and HIF2α. Folliculin was identified as a tumor suppressor gene. Its deletions and mutations are associated with a potential risk of renal cancer. At present, the specific molecular mechanism of FLCN-induced proliferation, invasion, and migration in ccRCC remains elusive.

**Methods:** Cell proliferation was measured by flow cytometry analysis, while cell migration and invasion were measured by wound healing assay and Matrigel invasion assay. The expression of FLCN, HIF2α, MMP9, and p-AKT was examined by Western blotting. The cells were transfected with plasmids or siRNA to upregulate or downregulate the expression of FLCN. Immunofluorescence microscopy was carried out to display the HIF2α location. We also determined the correlation of FLCN and HIF2α in human renal cancer samples.

**Results:** FLCN was combined with HIF2α in renal tubular epithelial and cancer cells, and it effectively alleviates the deterioration of renal cancer cells by degrading HIF2α. The silencing of FLCN showed a promotion of HIF2α protein expression via PI3K/mTORC2 pathway, which in turn led to an increase in downstream target genes *Cyclin D1* and *MMP9*. Moreover, interfering with siFLCN advanced the time of HIF2α entry into the nucleus.

**Conclusions:** Our study illustrated that FLCN could be a new therapeutic target in ccRCC. FLCN combined with HIF2α and identified a novel PI3K/mTORC2/HIF2α signaling in ccRCC cells.

## Introduction

Renal cell carcinoma is a malignant tumor originating in the renal tubular epithelial system, most of which are clear cell renal cell carcinoma (ccRCC, ~75%) (Kovacs et al., 1997). Cell viability is important for various physiological processes, such as embryonic development, angiogenesis, and tumor proliferation, invasion, and metastasis. Birt–Hogg–Dubé syndrome is caused by inactivating the mutations of FLCN, a tumor suppressor gene which encodes folliculin (Linehan et al., 2010). The common symptoms of Birt–Hogg–Dubé (BHD) syndrome are lung cyst, spontaneous pneumothorax, skin fibrofolliculomas, and renal cancer (Toro et al., 2008). It is also reported that the inactivation of FLCN is an initial step in the development of renal tumors in BHD (Hasumi et al., 2009). Work done by Sok Kean Khoo and Laura S. Schmidt has confirmed a tumor suppressor role for FLCN (Khoo et al., 2001; Schmidt et al., 2001; Schmidt and Linehan, 2015b). Similarly, the FLCN-deficient animal models (Baba et al., 2008) showed activated mTOR and its downstream pathway effectors (Hudon et al., 2010). This paper suggested that FLCN-deficient kidney tumors showed the activation of mTOR and AKT (Chen et al., 2008), and this regulation mechanism is also established in humans (Hartman et al., 2009; Linehan et al., 2010; Schmidt and Linehan, 2015a).

Hypoxia-inducible factor (HIF) is a crucial mediator of hypoxic adaptation. Previous studies have shown that the renal tumor-associated gene VHL plays a decisive role in regulating HIF expression (Gallou et al., 1999; Kondo and Kaelin, 2001). Similarly, research data also show that there are many links between FLCN and HIF1α (Baba et al., 2008; Aleksic et al., 2016). Our pretest result indicated that the *FLCN* gene acts as a regulator of renal tumors, and its knockdown resulted in a significant increase in HIF2α protein levels. *Cyclin D1* and *MMP9* have been identified as HIF2α-regulated genes (Yang et al., 2010; Preston et al., 2011) which can mediate cancer cell proliferation and invasion (Maranchie and Zhan, 2005; Pawlus et al., 2012; Du et al., 2017).

In this study, we hypothesized that FLCN may regulate HIF2α through binding to HIF2α to suppress the expression of Cyclin D1 and MMP9 during cell proliferation and invasion. Here we showed that FLCN regulates the nuclear export timing of the transcription factor HIF2α by forming a complex with HIF2α. The knockdown of *FLCN* caused HIF2α to enter the nucleus in advance, further exacerbating the aggressive behavior of tumor. In addition, FLCN/HIF2α was also regulated by the PI3K/mTORC2 signaling pathway. These findings revealed a novel relationship between FLCN and HIF2α in clear cell renal cell carcinoma proliferation, invasion, and metastasis.

## Materials and Methods

### Ethics Statement

The studies involving human participants were reviewed and approved by the Ethics Committee of Nanjing Medical University. The patients/participants provided written informed consent to participate in this study.

### Cell Lines and Cell Culture

Normal renal tubular epithelial cell line HK-2 and human clear cell renal cell carcinoma lines 786-O and ACHN were obtained from the Cell Biology Institute of the Chinese Academy of Sciences (Shanghai, China).

The HK-2 and ACHN cells were cultured in Dulbecco's modified eagle medium (DMEM) (Hyclone, ThermoScientific, Waltham, MA, USA), supplemented with 10% fetal bovine serum (Gibco, Carlsbad, CA, USA) at 37°C in 5% CO_2_. The 786-O cells were cultured in RPMI 1640 (Hyclone, ThermoScientific, Waltham, MA, USA), supplemented with 10% fetal bovine serum at 37°C in 5% CO_2_. The cells were grown on 10-cm dishes for subsequent experiments.

### Plasmids and siRNAs

The full-length Epas1 plasmid was purchased from Youbio (Hunan, China). The pCMV-FLAG-FLCN plasmid was constructed as previously reported, using the following primer set. The above constructs were confirmed via DNA sequencing. The cells were seeded in a six-well plate, cultured to 80–90% confluence, and then transfected with Epas1 and pCMV-FLAG-FLCN plasmids, respectively, by using FuGENE HD Transfection Reagent (Promega Corporation, Madison, WI, USA).

The non-specific control siRNA and siRNAs for FLCN and EPAS1 were purchased from GenePharma (Shanghai, China). The cells were transfected with siRNA Duplex oligonucleotides using Lipofectamine 2000 (Invitrogen), according to the transfection method provided by the manufacturer. The siRNAs are listed in Table 1.

**Table 1:** siRNA sequences used for transfection.

**Target**	**Sequence**
siFLCN-1#	5′-GAUAAAGAGACCUCCAUUA-3′
siFLCN-2#	5′-GGAUCUACCUCAUCAACUCCUGGCC-3′
siEPAS1-1#	5′-GGAGCUAACAGGACAUAGUTT-3′
siEPAS1-2#	5′-ACUAUGUCCUGUUAGCUCCTT-3′

### Reagents and Antibodies

Cyclohexamide (CHX) was purchased from Sigma (USA); MG-132, MK2206 (AKT inhibitor), KU0063794 (mTORC1 and mTORC2 inhibitor), rapamycin (mTORC1 inhibitor), and LY294002 (PI3K inhibitor) were purchased from ApexBio. JR-AB2-011 was purchased from MedChenExpress. The rabbit monoclonal antibodies against AKT, phospho-AKT (Thr308, Ser473), Histone 3, MMP9, and HIF2α were purchased from Cell Signaling Technology. The rabbit monoclonal antibodies against RICTOR were purchased from Proteintech. The mouse monoclonal antibody against HIF2α was purchased from Millipore for immunofluorescence. The FLCN rabbit pAb was purchased from Cell Signaling Technology for Western blotting and from ABcam for immunoprecipitation. The GAPDH rabbit mAb and β-actin mouse mAb antibodies were purchased from Santa Cruz Biotechnology. The horseradish peroxidase-conjugated secondary antibodies were purchased from Jeckson Immunoresearch Laboratories.

### Immunoprecipitation and Western Blotting

For Western blotting, equal amounts of protein were run on sodium dodecyl sulfate-polyacrylamide gel electrophoresis (SDS-PAGE) and transferred to a nitrocellulose membrane. The resulting blots were blocked with 5% non-fat milk (in Tris-buffered saline with Tween 20) and incubated with primary antibodies overnight at 4°C. Then, protein bands were detected by incubating with secondary antibodies for 1–2 h at room temperature and visualized with enhanced chemiluminescence reagent (Millipore, Billerica, MA, USA) by Chemi Doc XRS + gel imaging system (Bio-Rad, USA). Densitometry analysis was performed using Quantity One software, and the band intensities were normalized to those of β-actin and GAPDH.

Immunoprecipitation was performed as previously described (Zhu et al., 2013). Briefly, the cell lysates were centrifuged to remove the cell debris and then were incubated with beads (Abmart) for 1–2 h. Endogenous FLCN and HIF2α were immunoprecipitated using an anti-FLCN or anti-HIF2α polyclonal antibody. The beads were boiled after extensive washing, resolved via SDS-PAGE, and analyzed via immunoblotting. The protein concentration was detected using the Odyssey system.

### Cytoplasmic and Nuclear Protein Extraction

Cytoplasmic and nuclear proteins were obtained using the Nuclear and Cytoplasmic Protein Extraction Kit (Beyotime) according to the manufacturer's instructions. The cells were harvested by centrifugation and resuspended in cytoplasmic extraction agent A. The solution was vigorously vortexed and then incubated on ice for 10 min. Then, cytoplasmic extraction agent B was added to the cell pellet. The pellet was vortexed and incubated on ice for 1 min. The pellet was vortexed again and centrifuged for 5 min at 5,000 rpm. The supernatant was collected as a cytoplasmic extract. The insoluble (pellet) fraction was suspended by a nuclear extraction agent. After having been vortexed several times, the mixture was centrifuged for 10 min at 12,000 rpm. The supernatant was collected as a nuclear extract.

### Real-Time Quantitative PCR

Total RNA was extracted using Trizol reagent (Invitrogen) and reversely transcribed with HiScript®Q RT SuperMix for qPCR (Vazyme, Nanjing, China) according to the protocol. Real-time PCR analyses were performed with AceQ® qPCR SYBR® Green Master Mix (High ROX Premixed) (Vazyme) on ABI StepOne™ Real-Time PCR System (Applied Biosystems, Foster City, CA, USA) at the recommended thermal cycling settings: one initial cycle at 95°C for 10 min, followed by 40 cycles of 15 s at 95°C and 60 s at 60°C. The gene expression levels were calculated with Rt (2^−ΔΔCT^) values by StepOne Software v2.1 (Applied Biosystems). The primer sequences used in the qRT-PCR are listed in Table 2.

**Table 2:** Primer sequences used for qRT-PCR.

**Gene**	**Sequence**
GAPDH	5′-CATCAGCAATGCCTCCTGCAC-3′
	5′-TGAGTCCTTCCACGATACCAAAGTT-3′
β-Actin	5′-TGGTGATGGAGGAGGTTTAGTAAGT-3′
	5′-AACCAATAAAACCTACTCCTCCCTTAA-3′
FLCN	5′-TGCTCTCCTCAGAGTTTGCTG-3′
	5′-GTTGGTCAGAGCCGCTTCAA-3′
HIF2α	5′-GTCTGCAAAGGGTTTTGGGG-3′
	5′-TGTGAGGTGCTGCCACCAG-3′
Cyclin A	5′-GGATGGTAGTTTTGAGTCACCAC-3′
	5′-CACGAGGATAGCTCTCATACTGT-3′
Cyclin B	5′-TTGGGGACATTGGTAACAAAGTC-3′
	5′-ATAGGCTCAGGCGAAAGTTTTT-3′
Cyclin C	5′-CCTTGCATGGAGGATAGTGAATG-3′
	5′-AAGGAGGATACAGTAGGCAAAGA-3′
Cyclin D1	5′-CAATGACCCCGCACGATTTC-3′
	5′-CATGGAGGGCGGATTGGAA-3′
Cyclin D2	5′-ACCTTCCGCAGTGCTCCTA-3′
	5′-CCCAGCCAAGAAACGGTCC-3′
Cyclin D3	5′-TACCCGCCATCCATGATCG-3′
	5′-AGGCAGTCCACTTCAGTGC-3′
Cyclin E	5′-ACTCAACGTGCAAGCCTCG-3′
	5′-GCTCAAGAAAGTGCTGATCCC-3′
Cyclin F	5′-GGAAAGCGACAGGAGGACAG-3′
	5′-TGGCAGACGATCTCACTGGAA-3′
Cyclin G1	5′-GAGTCTGCACACGATAATGGC-3′
	5′-GTGCTTGGGCTGTACCTTCA-3′
Cyclin G2	5′-TCTCGGGTTGTTGAACGTCTA-3′
	5′-GTAGCCTCAATCAAACTCAGCC-3′
Cyclin H	5′-AGGCACTTGAACAGATACTGGA-3′
	5′-CCAATATGGGATAGCGGGTCT-3′

### Immunofluorescence Microscopy

For immunofluorescent staining, the cells were seeded on coverslips. The cells were transfected with or without siFLCN and FLCN plasmids for the indicated time, fixed in ice-cold 4% paraformaldehyde for 20 min at room temperature, rinsed with 3× phosphate-buffered saline (PBS) for 5 min, and permeabilized with 0.1% Triton X-100 before blocking in 1% BSA for 1 h at room temperature. The cells were incubated with primary antibodies at 4°C overnight, washed, and then incubated with Alexa- or FITC-coupled secondary antibodies for 1.5 h at room temperature in a moist chamber. After washing with PBS, the samples were mounted with DAPI Fluoromount G (Southern Biotech, Birmingham, AL, USA). The images were acquired using an Olympus BX51 microscope coupled with an Olympus DP70 digital camera and Carl ZEISS MicroImaging GmbH (Jena, Germany, 700). The images are representatives of three independent experiments.

### Migration and Matrigel Invasion Assays

For the wound healing assay, 786-O or ACHN cells were grown as described above and were plated in six-well plates on glass cover slips. At ~24 h later, when the confluence reached 95–100%, the cells were incubated overnight in DMEM, and wounding was performed by scraping through the cell monolayer with a 10-μl pipette tip. The medium and the non-adherent cells were removed, and the cells were washed twice with PBS. The images were collected at 0 h time point using an inverted microscope (Carl Zeiss Meditec, Jena, Germany). The Cells were permitted to migrate into the area of clearing for 6, 12, and 24 h in an incubator. Then, the cells were removed from the incubator, and images were taken using the same inverted microscope. Care was taken to align the scratch along the y axis of the camera so as to aid in the subsequent image quantification.

For the Matrigel invasion assay, the cell invasion assay was performed using Transwells (8 μm pore size, millipore) with the inserts coated with Matrigel (50 mg/ml, BD Biosciences). The 786-O and ACHN cells (1.0 × 10^5^ cells/well) were seeded in the upper chambers with 0.1 ml Matrigel and allowed to invade through the Matrigel for 12 and 24 h. The cells that remained on the membranes were fixed with 4% paraformaldehyde and stained with 0.5% crystal violet.

The cell pictures were taken with Nikon TS100 (Tokyo, Japan) and counted by Image J software. All the assays were performed at least three times.

### CCK-8 Assay and Flow Cytometry Analysis

The cells transfected with plasmids or siRNA were seeded at a density of 3.5 × 10^3^ cells per well into a 96-well plate. After having been cultured, the cells were washed and added with 10 μl Cell Counting Kit-8 (CCK-8) per 100 μl of culture medium, and the plate was incubated in the dark for 2 h, followed by measurement of absorbance value at 450 nm using a microplate absorbance reader (Bio-Tek, Elx800, USA). The fold growth was calculated as the absorbance of treated sample/control sample absorbance.

Cell cycle analysis was performed by flow cytometry. Briefly, the cells were harvested and fixed in 80% ice-cold ethanol overnight. Then, the cells were incubated with RNase A and propidium iodide staining solution at 37°C for 30 min in darkness. Subsequently, the stained cells were analyzed using flow cytometry.

### Immunohistochemistry

Renal cancer tissue microarrays were purchased from Shanghai Xinchao Biotechnology Co., Ltd. (Shanghai, China). A total of 75 cases of renal clear cell carcinoma samples and their corresponding paracancerous tissue samples were used for immunohistological staining in our study. Briefly, after microwave antigen retrieval, the microarray tissues were incubated with FLCN (Proteintech) or HIF2α antibody (Sigma) overnight at 4°C, followed by 1 h of incubation with a secondary antibody. The sections were developed in diaminobenzidine solution under microscopic observation and counterstained with hematoxylin. The immunohistochemical staining results were taken by using Axioskop 2 plus microscope (Carl Zeiss). FLCN and HIF2α immunostaining was scored by the immunoreactive score as described previously (Zhang et al., 2015).

### Statistical Analysis

Student's *t*-test and repeated-measures were used to analyze the differences between groups by using the SPSS statistical software program (version 19.0; SPSS, Chicago, IL, USA). Data are presented as mean ± SEM. Values of *P* < 0.05 were considered as statistically significant. All the experiments were repeated at least three times.

## Results

### FLCN Is Involved in the Proliferation of ccRCC Cells

**Figure 1:**
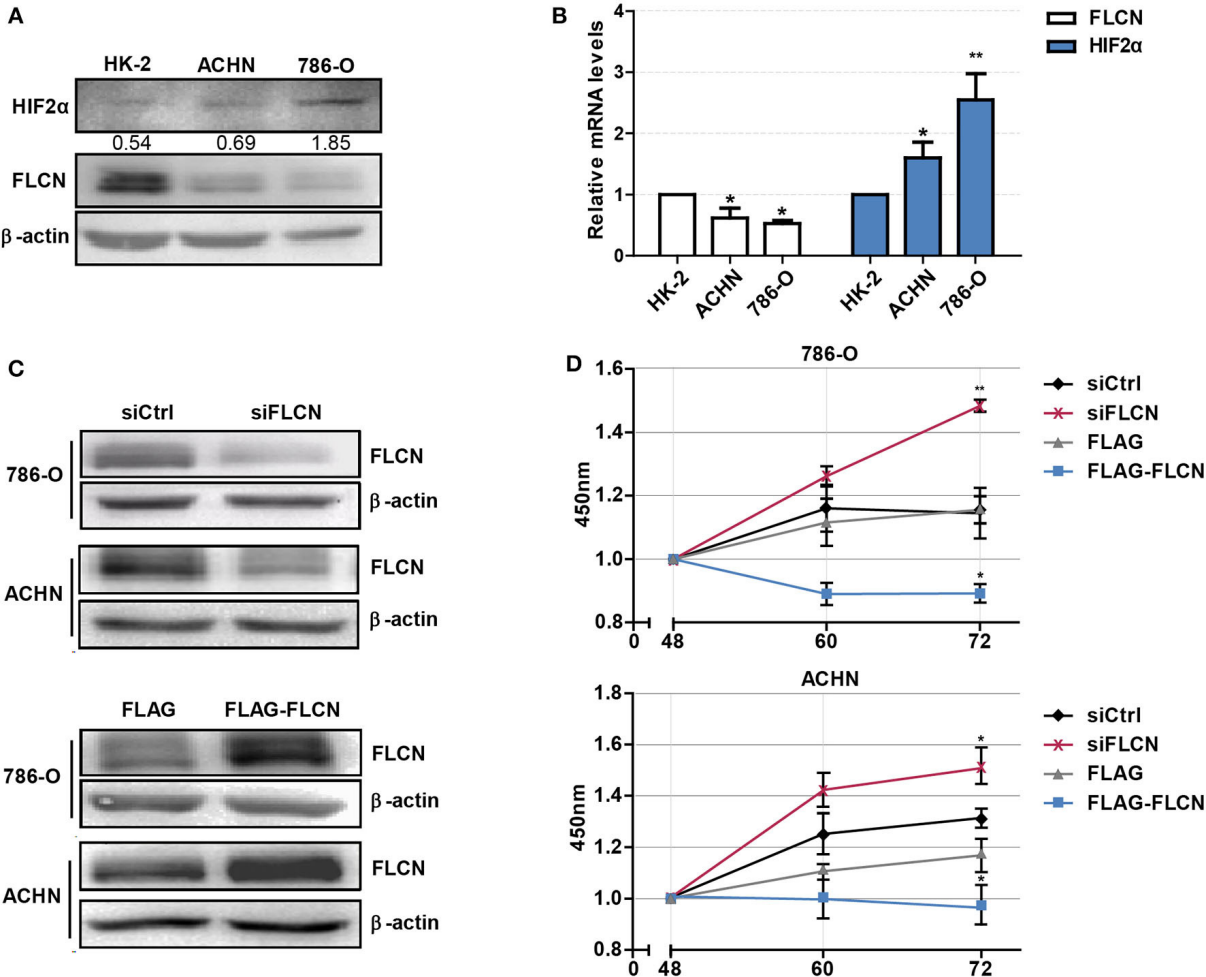
Folliculin (FLCN) knockdown accelerates the proliferation of clear cell renal cell carcinoma (ccRCC) cells. **(A)** Western blot detection of FLCN and HIF2α protein expression in ccRCC cell lines. **(B)** RT-qPCR analysis of FLCN and HIF2α mRNA expression in ccRCC cell lines. ccRCC cell lines, including 786-O and ACHN, were used in this experiment. The normal renal tubular epithelium cell line HK-2 was used as control. The 786-O and ACHN cells were transfected with FLCN siRNA or overexpression vector for 48 h, and **(C)** the protein expression of FLCN was confirmed by Western blot. **(D)** Cell proliferation was assessed by CCK-8 assay. The data are from three independently repeated experiments. *P < 0.05, **P < 0.01.

Firstly, we tested whether FLCN plays a crucial role in the HIF2α expression in ccRCC cells and verified the definite mechanisms involved. We first detected the protein levels of FLCN in normal renal tubular epithelial cells and clear cell renal cell carcinoma by Western blotting. The results showed that FLCN was poorly expressed in the 786-O and ACHN ccRCC cells compared to renal tubular epithelial cells HK-2. However, the HIF2α protein expression level was opposite to the FLCN trends (Figure 1A). The expression of FLCN and HIF2α had been examined by real-time PCR analysis in three cell lines (Figure 1B). Western blotting confirmed the siRNA-mediated specific knockdown of FLCN in 786-O and ACHN cells (Figure 1C), and the results of the CCK-8 assay showed that the knockdown efficiently promoted the cell viability (Figure 1D and Figure S1).

### FLCN Negatively Regulates ccRCC Cell Cycle and Invasion *in vitro*

**Figure 2:**
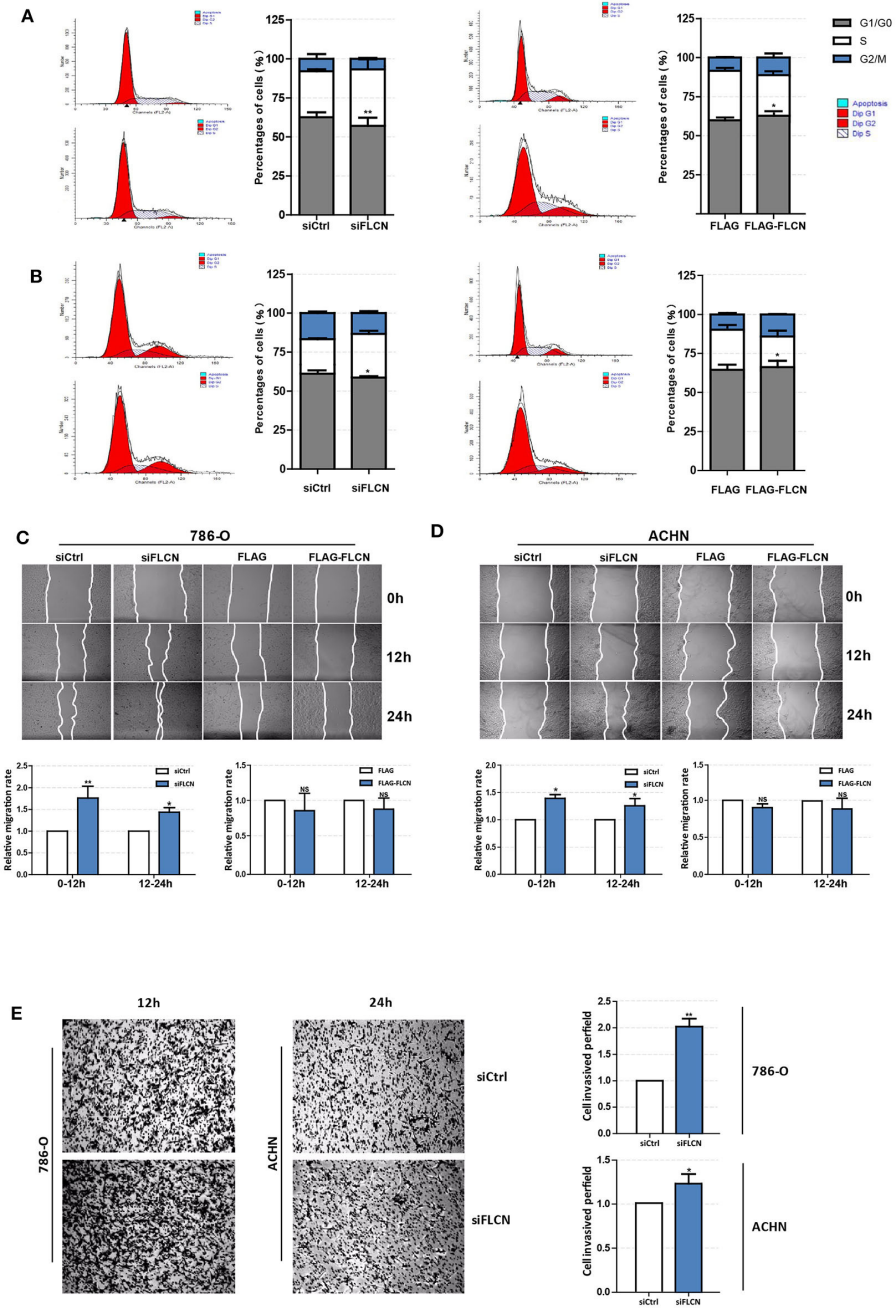
Folliculin (FLCN) regulates clear cell renal cell carcinoma cell cycle and invasion *in vitro*. **(A,B)** The effects of FLCN on 786-O **(A)** and ACHN **(B)** cell cycle distribution. The 786-O and ACHN cells were transfected with FLCN siRNA or overexpression vector for 48 h; flow cytometry assay was performed to test the cell cycle. **(C,D)** A representative of wound healing assays in 786-O **(C)** and ACHN **(D)** cells transfected with FLCN siRNA or overexpression vector for 12 or 24 h, and quantification of cell migration rate was performed. **(E)** The 786-O and ACHN cells were transfected with control siRNA or siFLCN for 12 or 24 h, and quantification of cell invasion rate was performed. The data are from three independently repeated experiments. *P < 0.05, **P < 0.01.

Since the FLCN knockdown increased the viability of the ccRCC cells, the second set of analyses examined the role of FLCN in cell motility. The results showed that the FLCN knockdown affected the cell cycle in 786-O cells and lead to a decrease in the proportion of cells in G1 phase and an increase in the proportion of cells in S phase (Figure 2A, left). In contrast, after transfection with the FLCN plasmid, we got the opposite results (Figure 2A, right). The above experiment was also performed with ACHN cells, and similar results were obtained (Figure 2B). The results of the wound healing assays showed that the rate of migration of the cells transfected with siFLCN #1 and #2 was increased when it was compared to the control group in 786-O cells (Figure 2C). The differences of the FLCN overexpression results were not significant. The same conclusion was also drawn in ACHN cells (Figure 2D). These results suggested a positive role for siFLCN in regulating ccRCC cell migration, and compared with long-term migration rate (24 h), the short-term (12 h) rate was more obvious. We checked cell invasion by transwell assay and found that the FLCN knockdown increased the invasion as compared with the control in 786-O and ACHN cells (Figure 2E).

### Cells Lacking FLCN Have Elevated Levels of HIF2α Expression

**Figure 3:**
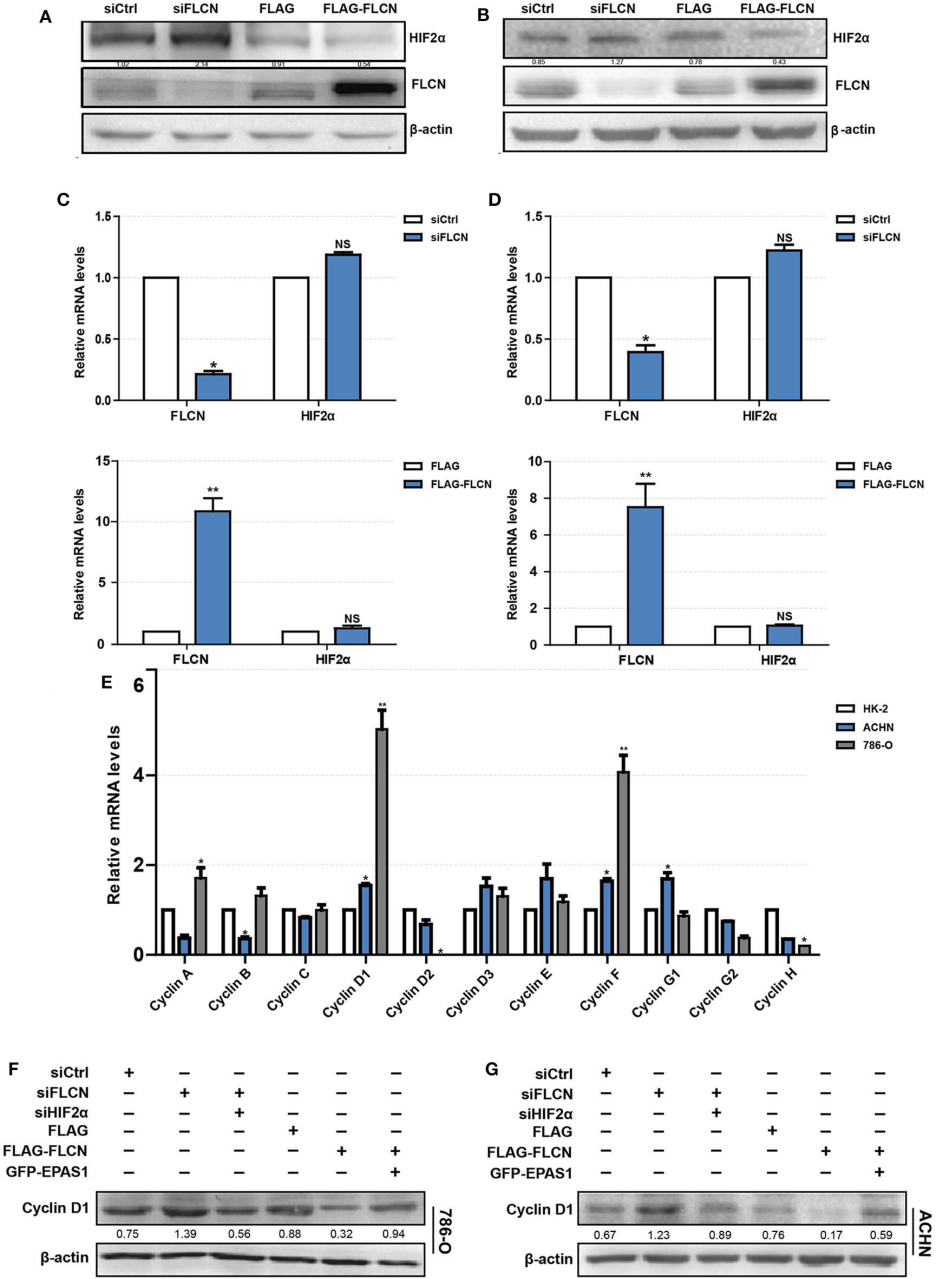
Folliculin (FLCN) regulates clear cell renal cell carcinoma (ccRCC) proliferation and cell cycle through HIF2α. **(A–D)** The regulatory effect of FLCN on HIF2α protein **(A,B)** and mRNA **(C,D)** in ccRCC cells was detected by Western blotting and RT-qPCR, respectively. The 786-O and ACHN cells were transfected with control siRNA or siFLCN for 48 h (protein) or 24 h (mRNA). **(E)** RT-qPCR analysis of cyclin family mRNA expression in ccRCC cell lines. ccRCC cell lines, including 786-O and ACHN, were used in this experiment. The normal renal tubular epithelium cell line HK-2 was used as control. **(F,G)** Western blotting analysis of cyclin D1 protein expression in 786-O **(F)** and ACHN **(G)** cell lines. The cells were co-transfected with siFLCN and siHIF2α or FLCN and EPAS1 overexpression plasmid for 48 h. The data are from three independently repeated experiments. *P <0.05, **P < 0.01.

To explore the mechanism of FLCN regulating cell proliferation and cell cycle, we focused on its putative interacting protein HIF2α. The FLCN knockdown or overexpression efficiency was determined by Western blotting. The results showed that the HIF2α and FLCN protein expressions are negatively correlated (Figure 3A). The conclusions in ACHN cells are consistent with the above findings (Figure 3B). In order to confirm HIF2α regulation by FLCN, the cells were treated with siFLCN or FLCN overexpression plasmids and then analyzed for FLCN and HIF2α mRNA level by real-time PCR. The results showed that the expression of *FLCN* gene was obviously changed with the treatment, but none of these programs caused a significant effect on HIF2α gene as compared with the control (Figures 3C,D). Then, the expression of the genes of cyclin family (FLCN/HIF2α downstream cell-viability-related genes) in HK-2 (normal), 786-O, and ACHN (ccRCC) cells was detected. Consistent with expectations, the expression level of cyclin D1 was significantly higher than the others (Figure 3E). According to this, the follow-up experiment used cyclin D1 as a marker to detect if HIF2α was directly downstream of FLCN. We co-transfected siFLCN/siHIF2α in 786-O cells and detected the *cyclin D1* protein (Figure 3F) and mRNA (Figure S2A, left) levels. Similarly, overexpression of FLCN reduced *cyclin D1*, and under these conditions, co-transfections with Epas1 plasmid rescued the cyclin D1 protein (Figure 3F) and mRNA (Figure S2A, right) levels. We also observed the same phenomenon in ACHN cells (Figure 3G and Figure S2B).

### FLCN Knockdown Restricts HIF2α Degradation of ccRCC Cells

**Figure 4:**
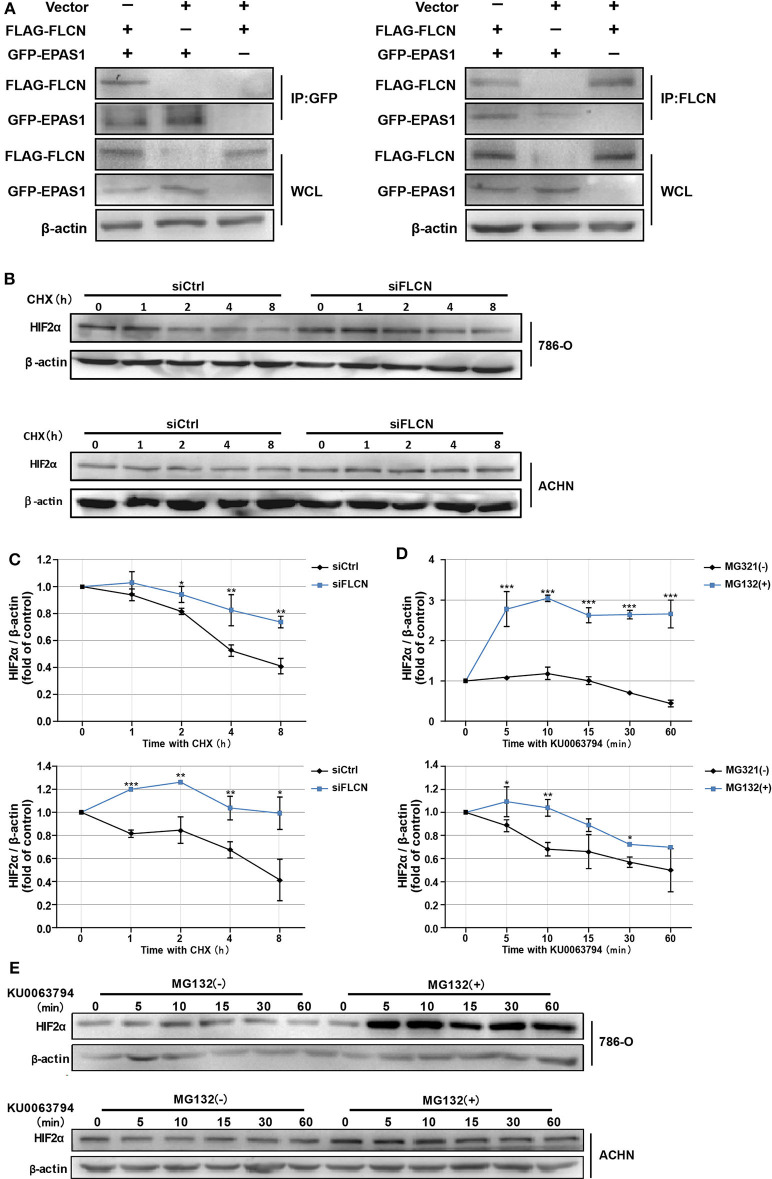
Folliculin (FLCN) knockdown restricts the HIF2α degradation of clear cell renal cell carcinoma cells. **(A)** Western blot showing FLAG-FLCN co-IP GFP-HIF2α or GFP-HIF2α co-IP FLAG-FLCN in ACHN cells. **(B,C)** The 786-O and ACHN cells were transfected with control siRNA or siFLCN for 48 h. In addition, its protein synthesis was blocked with cycloheximide (10 μg/ml) for the indicated times. Then, the cells were lysed and the HIF2α level was determined by Western blotting **(B)**. HIF2α bands were quantified and normalized against β-actin **(C)**. **(D,E)** The 786-O and ACHN cells added with its ubiquitination inhibitors dimethyl sulfoxide or MG-132 (5 μg/ml); the cells were then incubated with KU0063794 (10 ng/ml) for the indicated times. Then, the cells were lysed and the HIF2α level was determined by Western blotting **(D)**. The HIF2α bands were quantified and normalized against β-actin **(E)**. The data are from three independently repeated experiments. *P < 0.05, **P < 0.01, ***P < 0.001.

In order to confirm whether FLCN bound to HIF2α directly, we further identified the protein interaction in ACHN cells by co-immunoprecipitation (Figure 4A) and the endogenous protein interaction between renal cancer cells and normal renal tubular epithelial cells by immunoprecipitation (Figure S3). Whereas, 786-O/ACHN cells underwent a marked reduction or increase in FLCN, the abundance of HIF2α mRNA was not altered greatly (Figures 3C,D). Thus, we concluded that instead of a transcription-dependent mechanism, FLCN might modulate HIF2α expression by promoting its degradation process. We found that the FLCN knockdown inhibited the HIF2α protein degradation level, with CHX stimulation in 786-O (Figures 4B,C, top) and ACHN cells (Figures 4B,C, bottom). To explore the causes for the above phenomenon, the cells were treated with KU0063794 (mTOR inhibitor) for different times and then with or without ubiquitinated proteasome inhibitors to verify whether FLCN degraded HIF2α through the ubiquitination pathway. After treatment with KU0063794, the inhibition of mTOR was also shown to retard HIF2α degradation in 786-O (Figures 4D,E, top) and ACHN cells (Figures 4D,E, bottom).

### FLCN Knockdown Induces HIF2α Transportation to the Nucleus

**Figure 5:**
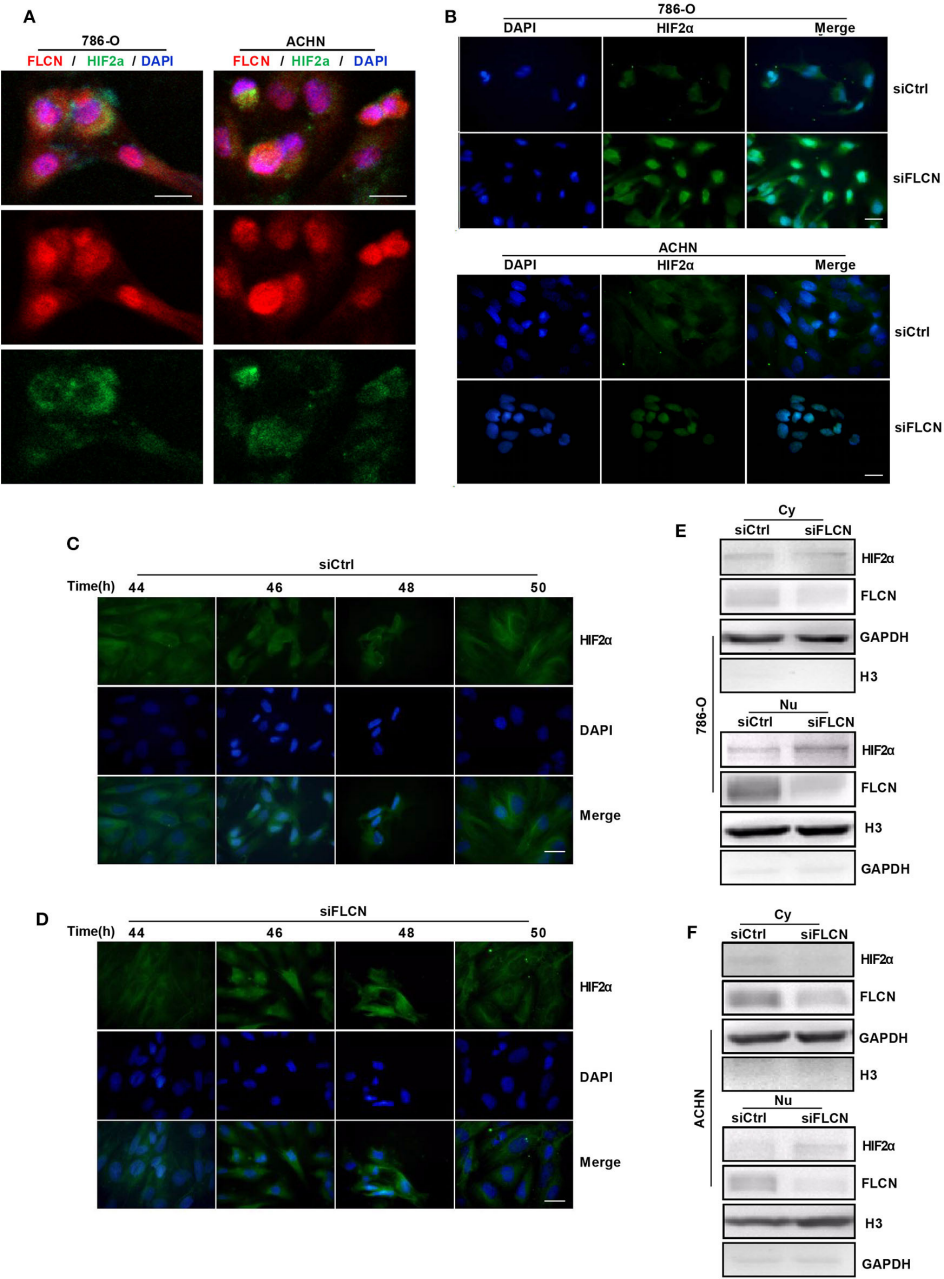
Folliculin (FLCN) knockdown induces HIF2α transportation to the nucleus. **(A)** Double immunofluorescence analysis was performed for FLCN and HIF2α. 4′,6-Diamidino-2-phenylindole (DAPI) fluorescence is shown as blue, FLCN immunofluorescence is shown as red, and HIF2α immunofluorescence is shown as green. **(B)** The 786-O and ACHN cells were transfected with control siRNA or siFLCN for 46 or 48 h. The HIF2α immunofluorescence is shown as green; the cell nucleus was labeled with DAPI. **(C–F)** The 786-O cells were transfected with control siRNA **(C)** or siFLCN **(D)** for the indicated times. HIF2α in 786-O cell immunofluorescence is shown as green; the cell nucleus was labeled with DAPI. The extracts of cytoplasm **(E)** and nucleus **(F)** were subjected to Western blotting to detect HIF2α. GAPDH or Histone 3 was used as control for the cytoplasm or nucleus part. Three independent experiments were carried out. Scale bar, 20 μm.

In view of the above findings, we further explored the influence of FLCN on the function of HIF2α in cell proliferation. Firstly, we examined the exact location of FLCN and HIF2α in the cells and found that these two molecules were co-localized (Figure 5A). Then, the immunofluorescence results showed that the knockdown of FLCN promoted HIF2α to enter the nucleus (Figure 5B). After 46–48 h (approximately the time of cell division generation) of FLCN knockdown, HIF2α turned massive in the 786-O cell nucleus at this time point (Figure 5D), compared to the control group (Figure 5C). Therefore, we verified the validity of this conclusion again by Western blotting in 786-O cells. The results showed visible nucleus HIF2α, which was further increased by FLCN knockdown for 46–48 h (Figure 5E, bottom), and a slight decrease in the expression of HIF2α in the cytoplasma (Figure 5E, top). The same experiment was also repeated in ACHN cells, and similar results were obtained (Figure 5F).

### HIF2α Mediates FLCN-Induced Cell Invasion via PI3K/mTORC2 Signaling Pathways

**Figure 6:**
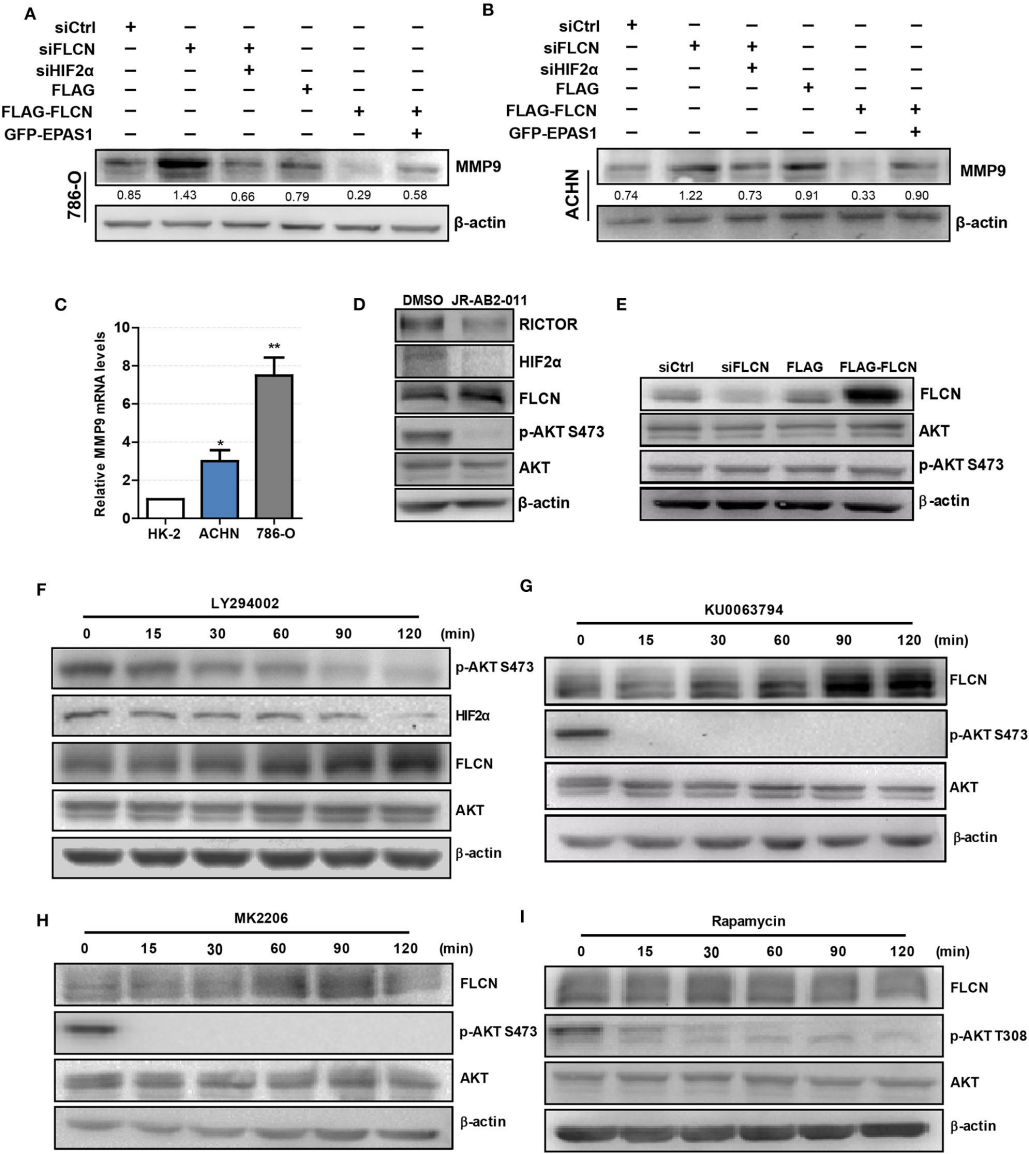
Folliculin (FLCN) regulates clear cell renal cell carcinoma (ccRCC) invasion via the PI3K/mTORC2/HIF2α signaling pathway. **(A,B)** Western blotting analysis of MMP9 protein expression in 786-O **(A)** and ACHN **(B)** cell lines. The cells were co-transfected with siFLCN and siHIF2α or FLCN and EPAS1 overexpression plasmid for 48 h. **(C)** RT-qPCR analysis of MMP9 mRNA expression in ccRCC cell lines. ccRCC cell lines, including 786-O and ACHN, were used in this experiment. The normal renal tubular epithelium cell line HK-2 was used as control. **(D)** The 786-O cells were incubated with JR-AB2-011 (RICTOR inhibitor, 2 μM) for 24 h; the protein levels of RICTOR, HIF2α, FLCN, P-AKT, and AKT were examined. **(E)** The 786-O cells were transfected with control siRNA or siFLCN; the protein levels of p-AKT were detected. **(F–I)** The 786-O cells were incubated with LY294002 (PI3K inhibitor, 10 μM), KU0063794 (mTORC inhibitor, 10 μM), MK2206 (AKT inhibitor, 5 μM), and rapamycin (mTORC1 inhibitor, 10 μM) for 0–120 min; the protein levels of FLCN, P-AKT, and AKT were examined. The data are from three independently repeated experiments. *P < 0.05, **P < 0.01.

We co-transfected siFLCN/siHIF2α in 786-O cells and detected the MMP9 protein levels. Similarly, the overexpression of FLCN reduced MMP9, and under these conditions, co-transfections with Epas1 plasmid rescued the MMP9 protein levels (Figure 6A). We also observed the same phenomenon in ACHN cells (Figure 6B). Then, we examined the MMP9 mRNA levels in HK-2, 786-O, and ACHN cells (Figure 6C).

Based on the above observations, our study hypothesized that FLCN relied on the PI3K/mTORC signaling pathway to achieve the regulation of HIF2α. Firstly, we treated the cells with JR-AB2-011 (RICTOR inhibitor), which promoted FLCN expression (Figure 6D and Figure S4). Then, we detected the expression of *FLCN* and found that the silencing or the overexpression of this gene had no effect on p-AKT S473, an indicator of mTORC2 activity (Figure 6E). These results indicate that FLCN may be downstream of mTORC2. Next, we treated 786-0 cells with LY294002, an inhibitor of PI3K, and found that LY294002 treatment decreased HIF2α expression (Figure 6F). In order to verify the clear upstream and downstream of FLCN, we treated the cells with KU0063794 (mTORC inhibitor) and MK2206 (AKT inhibitor), which promoted FLCN expression (Figures 6G,H). These results indicated that FLCN was involved in the signaling condition of mTORC2/HIF2α. Finally, we found that rapamycin stimulation did not affect the FLCN expression (Figure 6I), which further validated that mTORC2, but not mTORC1, could regulate HIF2a by controlling FLCN.

### FLCN Is Marginally Expressed in Human Renal Cancer Samples and Correlated With HIF2α Expression

**Figure 7:**
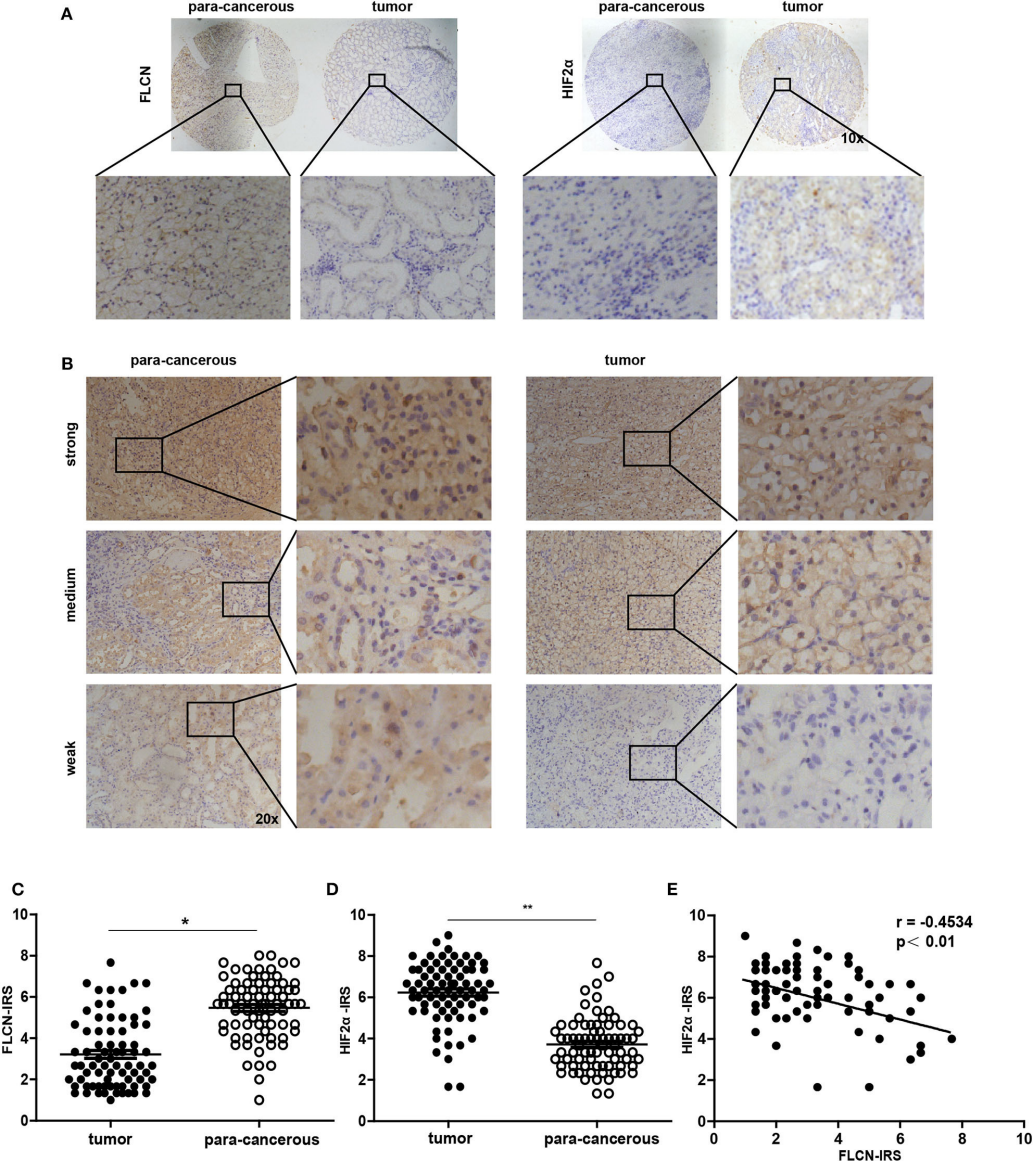
Analysis of folliculin (FLCN) and HIF2α expressions in renal cancer tissues. **(A)** Representative images of malignant renal cancer tissue and paracancerous tissue stained for FLCN and HIF2α are shown. **(B)** Representative images of FLCN in stained renal cancer tissues are shown. The positive staining of FLCN is shown in brown color and the cell nuclei were counterstained with hematoxylin. **(C,D)** Analysis for FLCN and HIF2α staining in renal cancer tissue (*n* = 75) and paracancerous tissue (*n* = 75) by immunoreactive scores. **(E)** Scatterplot of correlated protein levels between FLCN and HIF2α in renal cancer tissue (*n* = 75). *P < 0.05, **P < 0.01.

In order to investigate whether the *in vitro* experimental findings were consistent with the pathogenesis and the progression of renal cancer in humans, we examined the FLCN and the HIF2α expression patterns in renal cancers (75 paired cases) by a tissue microarray (Figure 7A). The immunohistochemistry results indicated that FLCN was marginally expressed in tumor tissues compared with matched para-cancerous tissues (Figure 7B), but HIF2α was reversed. The immunoreactive score was calculated as the intensity of the staining reaction multiplied by the percentage of positive cells. Based on the analysis of 75 groups of renal cancer tissues and para-cancerous tissues, we found that the FLCN expression level was low in tumor tissues, while the HIF2α expression level was in a reversed pattern (Figures 7C,D). Furthermore, immunostaining of FLCN and HIF2α in these 75 primary renal tumors revealed a negative correlation in expression (*r* = −0.4534, *P* < 0.01) (Figure 7E). Overall, the clinical data supported our *in vitro* results.

## Discussion

There are many reports on genes associated with kidney cancer pathogenesis (Blume-Jensen and Hunter, 2001; Zbar et al., 2003), including *VHL, FH, MET, FLCN*, etc. (Davis et al., 2014; Hsieh et al., 2017). The *FLCN* gene is a tumor suppressor (Nickerson et al., 2002), but the mechanism by which *FLCN* deficiency causes renal cancer is not completely understood. The PI3K/AKT/mTOR pathway drives cell proliferation, motility, and migration in a variety of malignancies (Blume-Jensen and Hunter, 2001; Vivanco and Sawyers, 2002). The loss of FLCN is linked with an activity of the AKT/mTORC pathway (Cash et al., 2011; Schmidt and Linehan, 2018), leading to Birt–Hogg–Dubé autosomal dominant syndrome and increasing the risk of hybrid oncocytic renal cell carcinoma (RCC) and pulmonary cysts (Kapitsinou and Haase, 2008; Bonora et al., 2015). It can be seen that these four genes are closely related to HIF and RCC. In summary, we decided to focus on FLCN and study the effects of its inactivation on ccRCC, HIF, and the related signaling pathways involved.

A current literature reports that there is controversy about the relationship between FLCN and HIFα (Hasumi et al., 2009). HIFα is highly expressed in BHD tumors, and FLCN knockout leads to an increased activity of HIFα. However, most of the studies just stayed in the phenomenon. The mechanism is not discussed and the specific relationship between FLCN and HIF1α/HIF2α is unclear. The main points are as follows: Preston et al. (2011) thought the absence of the FLCN gene did not affect the HIF1α levels under normoxia/hypoxia, and it is also mentioned that HIF2α is highly expressed under hypoxic conditions. In another report, the immunohistochemistry results indicate that FLCN deficiency increases HIF1α and its downstream expression (Hasumi et al., 2014). A document in the same year claimed that HIF1α increased in the lack of FLCN and promoted tumor growth (Khabibullin et al., 2014). In addition, our study showed that silencing of FLCN enhanced the expression of the HIF2α protein and implicated a novel molecular mechanism that FLCN interacted with HIF2α and impacted ccRCC physiological activity. We examined the FLCN and HIF2α expression patterns in renal cancers (75 paired cases) by a tissue microarray; FLCN and HIF2α showed a negative correlation. The clinical data supported our *in vitro* results. In the process, we also detected the mRNA level of HIF2α and found that there was no significant change, suggesting that FLCN did not regulate HIF2α at the transcriptional level but might control the degradation of HIF2α.

In the experiment, it could be clearly seen that the RNA and the protein levels of FLCN in ccRCC were relatively low compared to that of normal renal tubular epithelial cells. This again proved that *FLCN* acted as a tumor suppressor gene, but its role might be more prone to maintain the normal physiological function of the cells. The *FLCN* deletion activated the downstream signaling pathway, resulting in tumor formation rather than saving the deterioration of existing tumor cells. Based on the above observations, we transfected siFLCN with ccRCC to demonstrate that FLCN deficiency had a detailed mechanism for inducing renal cell carcinoma. At the same time, FLCN overexpression experiments were also performed to verify whether the increasing FLCN expression levels in renal tumors caused by other factors contributed to the alleviation of tumor cell deterioration. In the experiment, we selected two ccRCC cell lines 786-O and ACHN, of which 786-O was a cell line with VHL gene deletion, and HIF2α was still highly expressed under normoxia, which was more convenient for us to observe the results. Then, we added CHX interfering protein translation to the cell line knocked down by FLCN. The results showed that, compared with the control group, the content of HIF2α in the FLCN interference group could be maintained at a relatively stable level within 0–8 h after the addition of CHX, indicating that FLCN might accelerate the degradation of HIF2α protein. We hypothesized that this mechanism might have some similarities with VHL ubiquitination and the degradation of HIF1α (Epstein et al., 2001; Ivan et al., 2001; Jaakkola et al., 2001). To further verify the specific mechanism of this degradation, we added the mTORC inhibitor to upregulate the FLCN and, at the same time, added the proteasome inhibitor MG-132 to inhibit the ubiquitination degradation pathway. The results showed that, although increased FLCN could reduce HIF2α, after adding MG132 the expression of HIF2α was significantly increased, which indicated that FLCN was likely to achieve the degradation of HIF2α by ubiquitination, thereby inhibiting the proliferation of tumors.

In addition, during the experiment, we also unexpectedly found that the transcription factor HIF2α in the siFLCN group increased significantly in the nucleus at 46–48 h compared with the control group. In order to study the occurrence of this phenomenon, it was caused by the FLCN knockdown which promoted the acceleration of HIF2α into the nucleus rather than the increase of the total protein of HIF2α. We transfected siFLCN in ccRCC and observed the cell morphological phenomenon from 0 h with immunofluorescence and then detected the distribution of HIF2α in the cells every 2 h (only the distribution of at 44–50 h was shown in the figures). The results showed that HIF2α, in the siFLCN group of the 786-O cell line, began to enter the nucleus in 46–48 h, rather than gradually increasing with time. At the same time, the distribution and the content of HIF2α in the nucleus of the control group did not change significantly. This indicated that FLCN could not only bind with and degrade HIF2α but also participate in the regulation of HIF2α nuclear import. We then explored the mechanism of how PI3K/mTORC2 signaling regulated the invasion of FLCN. Recent studies have shown that mTORC is activated in kidney tumors that lack FLCN (Chen et al., 2008). Similarly, Hartman et al. reported that the downregulation of BHD reduced the TORC1 activity (Bonora et al., 2015), which suggested a close relationship between FLCN and mTORC. It was also reported that mTORC promoted HIF expression, mTORC2 focused on HIF2α, and HIF1α was sensitive to rapamycin but HIF2α was tolerant (Toschi et al., 2008). So, we focused on the regulation mechanism of FLCN in the PI3K/mTORC2/HIF2α signaling pathway. In summary, by silencing FLCN, HIF2α might move into the nucleus and activate downstream-related tumor signaling pathways.

In conclusion, we demonstrate the specific mechanism by which FLCN regulates HIF2α-induced renal cancer cell proliferation, migration, and invasion. FLCN interacts with HIF2α and promotes HIF2α degradation in human renal cancer cells. Although the current study has contributed to the mechanistic understanding of the role of FLCN in regulating renal cancer cell physiological activities, the issue as to how FLCN precisely adjusts HIF2 degradation in ccRCC cells is unlikely to be settled in this paper. Our findings point out that FLCN/HIF2α expression may be a novel therapeutic target for preventing renal cancer proliferation and invasion.

## Data Availability Statement

All datasets generated for this study are included in the article/Supplementary Material.

## Ethics Statement

The studies involving human participants were reviewed and approved by the Ethics Committee of Nanjing Medical University. The patients/participants provided written informed consent to participate in this study.

## Author Contributions

LG, YZ, and XZ designed the study. XZ, JC, and YZ performed the experiments. XZ, YM, HZ, LL, YW, PM, and LZ performed the statistical analysis. XZ, YC, and YZ drafted the manuscript. LG, YZ, and JD supervised the experimental work. All authors contributed to the article and approved the submitted version.

## Conflict of Interest

The authors declare that the research was conducted in the absence of any commercial or financial relationships that could be construed as a potential conflict of interest.

## Supplementary Material

Figure S1 Folliculin knockdown accelerates the proliferation of clear cell renal cell carcinoma cells. Cell proliferation was assessed by CCK-8 assay. The data are from three independently repeated experiments. ^*^*P* < 0.05.

Figure S2 Folliculin (FLCN) regulates Cyclin D1 through HIF2α. **(A,B)** RT-qPCR analysis of cyclin D1 mRNA expression in 786-O **(A)** and ACHN **(B)** cell lines. The cells were co-transfected with siFLCN and siHIF2α or FLCN and EPAS1 overexpression plasmid for 24 h. The data are from three independently repeated experiments. ^*^*P* < 0.05, ^**^*P* < 0.01.

Figure S3 Endogenous binding of folliculin (FLCN) and HIF2α. Western blot showing FLCN co-IP HIF2α or HIF2α co-IP FLCN in 786-O, ACHN, and HK-2 cells. IgG was used as control.

Figure S4 RICTOR reduces folliculin expression. The 786-O cells were incubated with JR-AB2-011 (RICTOR inhibitor, 2 μM) for 1–120 min. The protein levels of RICTOR, FLCN, P-AKT, and AKT were examined.
