# Supplementary figures and images for: FLCN Regulates HIF2α Nuclear Import and Proliferation of Clear Cell Renal Cell Carcinoma
Source: Front Mol Biosci. 2020 Jul 28;7:121. doi: 10.3389/fmolb.2020.00121 (PMC7399043; doi:10.3389/fmolb.2020.00121)

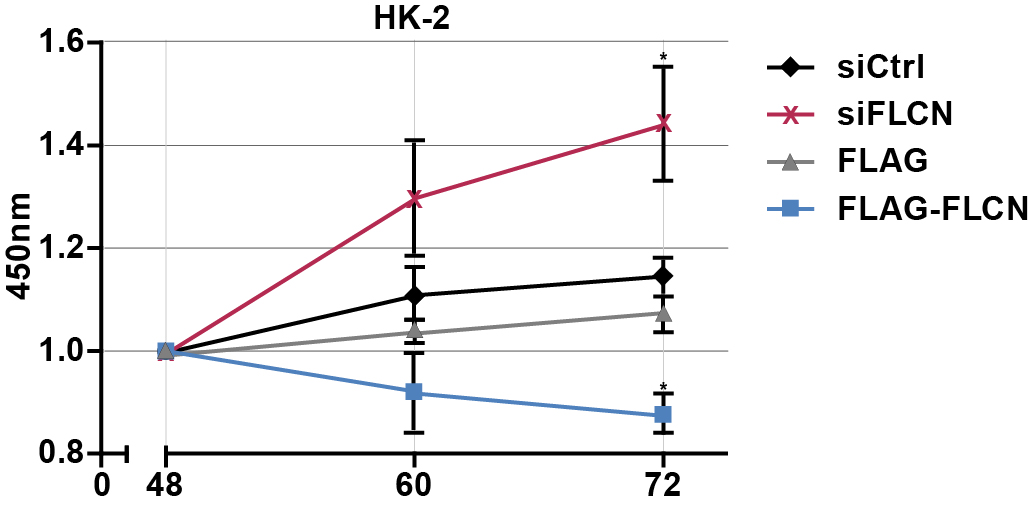

Supplement: Figure S1 — Folliculin knockdown accelerates the proliferation of clear cell renal cell carcinoma cells. Cell proliferation was assessed by CCK-8 assay. The data are from three independently repeated experiments. *P < 0.05. [file Image_1.jpg]

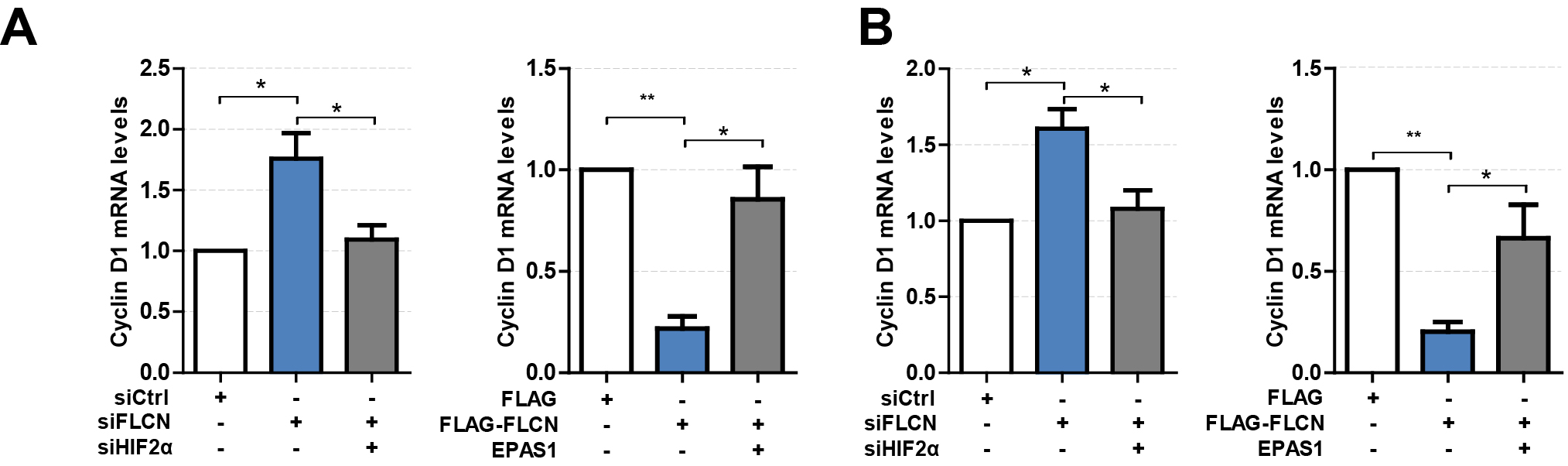

Supplement: Figure S2 — Folliculin (FLCN) regulates Cyclin D1 through HIF2α. (A,B) RT-qPCR analysis of cyclin D1 mRNA expression in 786-O (A) and ACHN (B) cell lines. The cells were co-transfected with siFLCN and siHIF2α or FLCN and EPAS1 overexpression plasmid for 24 h. The data are from three independently repeated experiments. *P < 0.05, **P < 0.01. [file Image_2.JPEG]

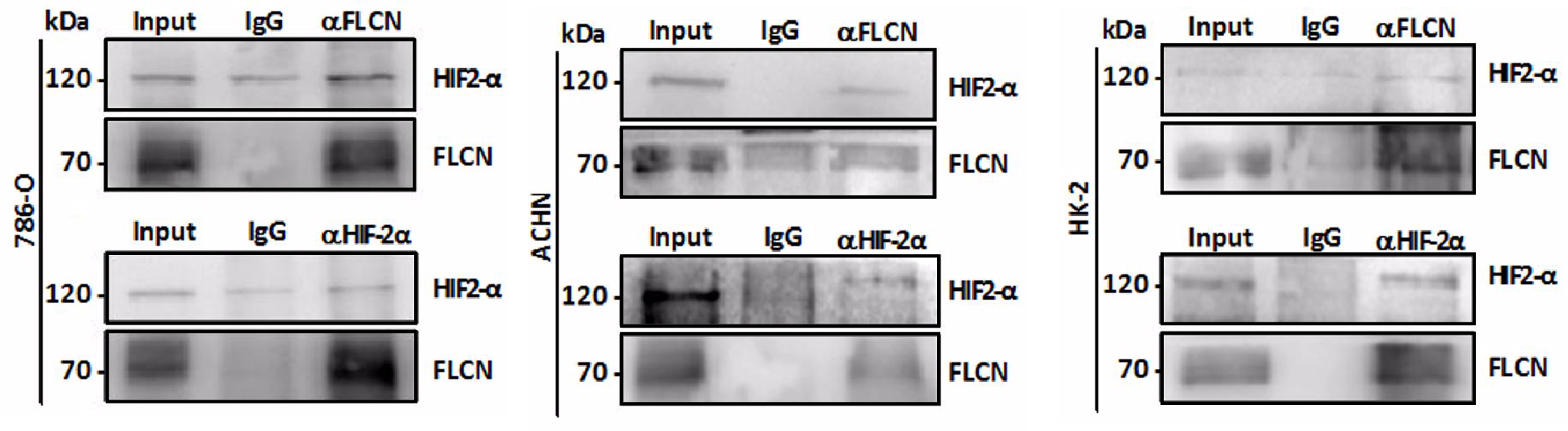

Supplement: Figure S3 — Endogenous binding of folliculin (FLCN) and HIF2α. Western blot showing FLCN co-IP HIF2α or HIF2α co-IP FLCN in 786-O, ACHN, and HK-2 cells. IgG was used as control. [file Image_3.jpg]

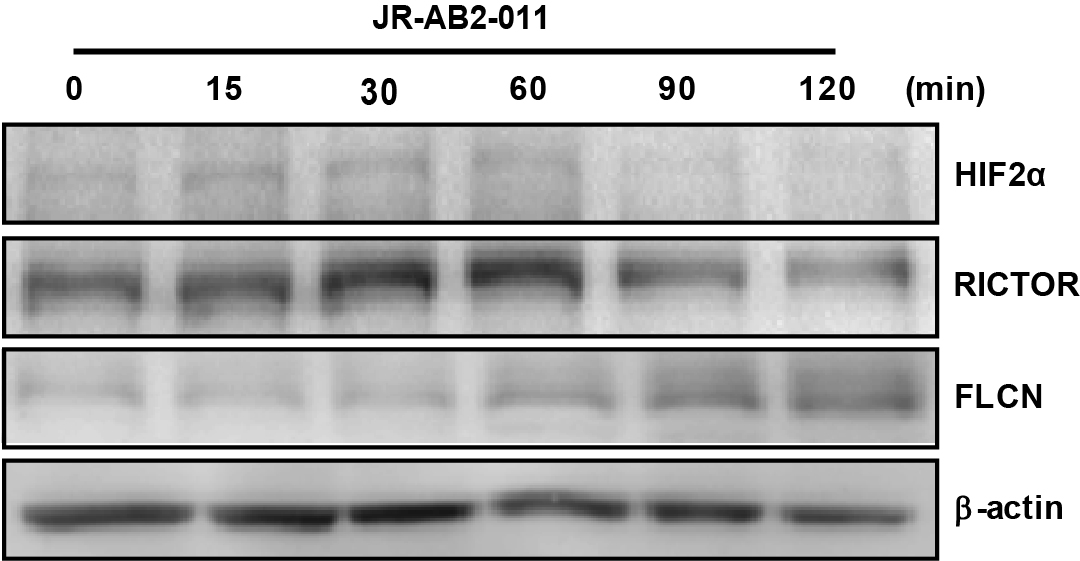

Supplement: Figure S4 — RICTOR reduces folliculin expression. The 786-O cells were incubated with JR-AB2-011 (RICTOR inhibitor, 2 μM) for 1–120 min. The protein levels of RICTOR, FLCN, P-AKT, and AKT were examined. [file Image_4.jpg]
